# A Fresh Perspective on the Internal Plasticizing Effect in Non-Polymeric Glass-Formers

**DOI:** 10.1021/acs.jpcb.5c01809

**Published:** 2025-05-30

**Authors:** Andrzej Nowok, Hubert Hellwig, Piotr Kuś

**Affiliations:** † Department of Experimental Physics, 49567 Wrocław University of Science and Technology, Wybrzeże Wyspiańskiego 27, 50-370 Wrocław, Poland; ‡ Center for Integrated Technology and Organic Synthesis (CiTOS), MolSys Research Unit, 608565 University of Liège, B6a, Room 3/19, Allée du Six Août 13, 4000 Liège, Sart Tilman, Belgium; § Institute of Chemistry, University of Silesia, Szkolna 9, 40-006 Katowice, Poland

## Abstract

The internal plasticizing effect occurs when a flexible substituent, especially one linked to a rigid moiety, lowers the glass transition temperature (*T*_g_) of an organic compound by disrupting molecular packing and increasing the internal movement. While this concept has long been recognized in polymers, its potential application to low-molecular-weight, nonpolymeric compounds has gained attention only recently. In this study, we provide direct evidence supporting this concept among low-molecular-weight glass-formers by comparing the thermal and dynamic properties of structurally related 1,2-bis­(2-halogenethylthio)-4-methylbenzene and ester derivatives of 1,2-bis­(2-hydroxyethylthio)-4-methylbenzene. We show that increasing molar mass through a simple atom substitution without altering intermolecular interactions, such as exchanging chlorine for bromine, decelerates molecular dynamics in the liquid phase and raises *T*_g_ according to the typical dependence *T*_g_ ∼ *M* ^α^. This behavior arises from nearly identical conformational transformations in these compounds, where comparable energy barriers and intramolecular dynamics render molar mass the primary determinant of structural relaxation times. In contrast, similar increase in molar mass achieved by elongating the flexible substituent lowers the *T*_g_ value and enhances both the intra- and intermolecular dynamics, underscoring internal molecular flexibility and conformational diversity as a crucial factor controlling the glass transition and near-glass-transition phenomena. Our findings, supported by differential scanning calorimetry, broadband dielectric spectroscopy, and density functional theory calculations, indicate internal plasticization as a fundamental mechanism shaping the properties of molecular glass-formers.

## Graphical abstract

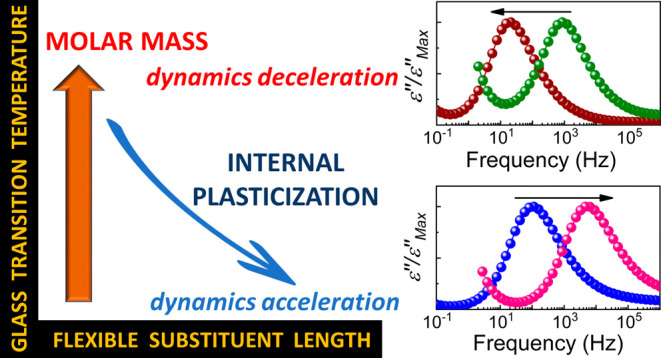

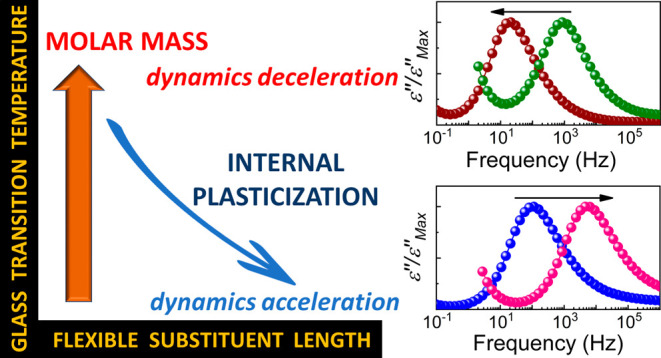

## 1 Introduction

The glass transition temperature, *T*_g_, is a point at which molecular motion in a liquid slows down so much that it begins to behave like an amorphous solid (glass).^1^ While this concept is well understood, predicting the ability of low-molecular-weight compounds to form glass and determining their *T*_g_ values based on solely molecular structure remains a challenge.^2^

Over the years, scientists have developed several models that enable such estimations, with one of the most widely accepted being the Boyer–Beaman rule. This rule proposes a linear correlation between *T*_g_ and the melting point, *T*_m_, of molecular glass-formers, with significant scatter in the experimental data and the *T*_g_/*T*_m_ ratio averaging around 0.7 across a broad range of substances.^3–5^ However, the melting point values are not always accessible as some organic low-molecular-weight organic compounds do not crystallize.^5^ Further investigations by DeRieux et al. and Li et al. have demonstrated that *T*_g_ is influenced by atomic composition and functional groups.^6,7^ Indeed, each added hydroxyl (−OH) group (up to eight groups) increases *T*_g_ by about 30 K, while carboxyl (−COOH) substituents have an even greater effect.^8,9^ These findings highlight that intermolecular forces, such as hydrogen bonding, dipole–dipole interactions, London dispersion forces, and ionic interactions, play a crucial role in determining *T*_g_.^8^ Recent studies using recurrent neural network (RNN) analysis reinforce these findings, identifying hydrogen-bonding ability, molecular rigidity, the presence of bulky aromatic rings, and flexible linear chains with weak intermolecular forces as key factors influencing *T*_g_.^10^ These studies also highlight that, beyond chemical composition, molar mass, *M*, plays a significant role.^10^

In this context, Novikov and Rössler demonstrated that *T*_g_ of nonpolymeric glass-formers generally increases with rising *M* according to the relation: *T*_g_ ∼ *M* ^α^, where α is an empirical power coefficient.^5^ However, this correlation does not hold universally, and the experimental *T*_g_ data often show deviations of up to 100 K at a given mass.^5^ Recent studies on ester derivatives of 1,2-bis­(2-hydroxyethylthio)-4-methylbenzene have revealed that even an opposite trend is possible among nonpolymeric glass-formers: in some cases, *T*_g_ decreases as *M* increases.^11^ This effect is particularly evident when a flexible functional group is attached to a rigid molecular core. It has been suggested that the flexible moiety acts in such systems as an internal plasticizer, increasing molecular flexibility and conformational diversity.^11,12^ According to this concept, the range of molecular motions increases (including end-group, subgroup, and crankshaft movements) as the length of the flexible moieties grows.^11,13^ This in turn disrupts their effective intermolecular alignment and expands free volume (understood in that studies in the most general sense as the unoccupied space between molecules,^11^ without invoking more precise definitions found in the literature^14^), ultimately facilitating and accelerating their motion. Consequently, the enhanced intramolecular flexibility allows for easier mutual rearrangements of entire molecules in the liquid phase, reducing the viscosity-related structural relaxation times at a given temperature and lowering the *T*_g_ value.^11^

In this study, we provide direct evidence supporting this concept among low-molecular-weight glass-formers. By comparing the thermal and dynamic properties of structurally similar halogenated and ester derivatives of 1,2-bis­(2-hydroxyethylthio)-4-methylbenzene, we demonstrate that increasing the molar mass through a simple atomic substitution (i.e., without altering intermolecular interactions) decelerates molecular dynamics in the liquid phase and raises *T*_g_ according to the typical dependence *T*_g_ ∼ *M* ^α^. In turn, a similar increase in molar mass achieved by elongating a flexible substituent lowers the *T*_g_ value and enhances both the intra- and intermolecular dynamics. We explore the underlying mechanisms behind these opposing trends and indicate that beyond molar mass, internal molecular flexibility, and conformational diversity are key factors governing the glass transition temperature. Our study is based on the combination of the density functional theory (DFT) calculations with various experimental techniques, such as broadband dielectric spectroscopy and differential scanning calorimetry (DSC).

## 2 Materials and Methods

### 2.1 Materials

The compounds under investigation are (1,2-bis­(2-chloroethylthio)-4-methylbenzene (compound **1**) and 1,2-bis­(2-bromoethylthio)-4-methylbenzene (compound **2**). They are commercially unavailable and were synthesized from 1,2-bis­(2-hydroxyethylthio)-4-methylbenzene. This substance was prepared by the alkylation reaction of 4-methylbenzene-1,2-dithiol with 2-bromoethanol in an aqueous solution of sodium hydroxide. The halogen derivatives **1** and **2** were obtained by the substitution reaction of the hydroxyl groups with SOCl_2_ or PBr_3_ according to the methodology described in the Supporting Information. The reagents were used without any preliminary purification. The purity of compounds **1** and **2** was confirmed by ^1^H and ^13^C NMR spectroscopy. The obtained results for compounds **1** and **2** were compared to those for the acetyl (compound **3**), propionyl (compound **4**) and butyryl (compound **5**) ester derivatives of 1,2-bis­(2-hydroxyethylthio)-4-methylbenzene which were partially reported previously.^11^ The chemical structures of compounds **1–5** are presented in Figure 1a.

**Figure 1:**
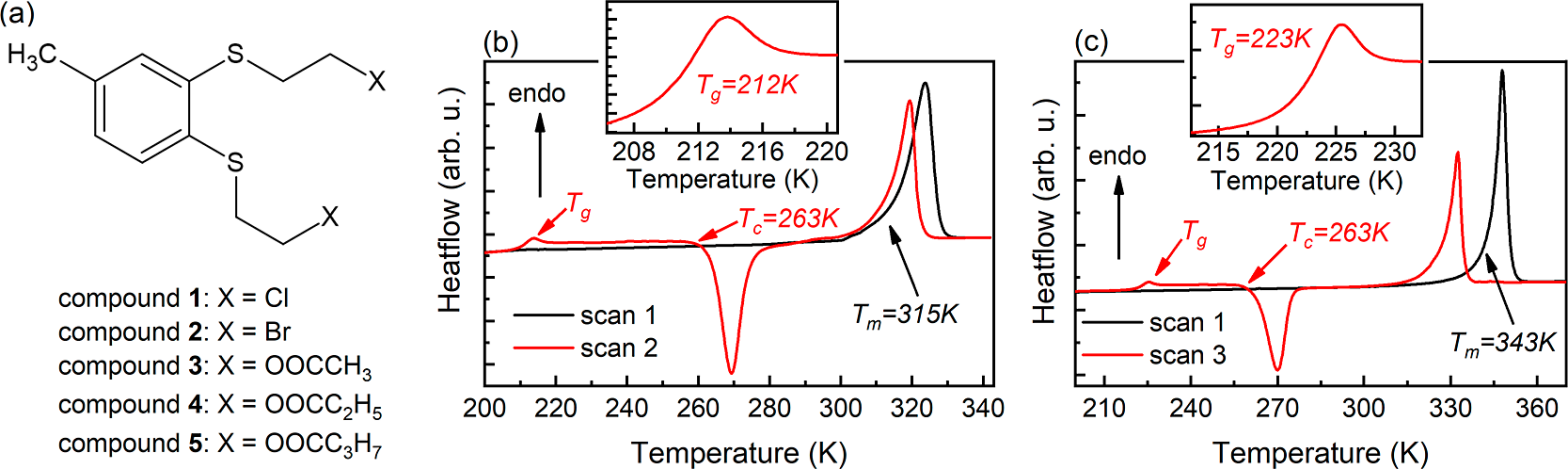
(a) Chemical structure of compounds **1–5**. (b) Two subsequent heating thermograms for compound **1** collected at a rate of 10 K/min; the inset highlights the glass transition. (c) Comparison of the initial and final heating thermograms for compound **2**, obtained at 10 and 15 K/min, respectively. The inset shows the thermal effect related to the glass transition.

### 2.2 NMR Spectroscopy

The ^1^H and ^13^C NMR spectra for compounds **1** and **2** were measured by using a Bruker Avance 400 MHz spectrometer (Bruker, Rheinstetten, Germany). Deuterated chloroform (CDCl_3_) or dimethyl sulfoxide (DMSO-*d*_6_) purchased from Cambridge Isotope Laboratories (Tewksbury, MA, USA) were used as the solvent. The chemical shifts were calibrated against the residual solvent signals, with CDCl_3_ peaks observed at 7.28 ppm for ^1^H NMR and 77.04 ppm for ^13^C NMR, or DMSO-*d*_6_ peaks observed at 2.50 ppm for ^1^H NMR and 39.52 ppm for ^13^C NMR. The collected spectra of compounds **1** and **2** are depicted in Figures S1–S4 in Supporting Information.

### 2.3 Thermogravimetry (TGA)

The thermal stability of compounds **1** and **2** was evaluated through thermogravimetric analysis (TGA) using a PerkinElmer Pyris 1 instrument under a nitrogen flow. During this experiment, the samples were subjected to heating from room temperature to 900 °C with a heating rate set at 15 K/min. The obtained results are presented in Supporting Information as Figures S5 and S6.

### 2.4 Differential Scanning Calorimetry (DSC)

Thermal properties of compounds **1** and **2** were analyzed using DSC techniques and a Mettler-Toledo DSC 1 STARe setup (Mettler Toledo, Columbus, OH, USA), equipped with an intracooler and an HSS8 ceramic sensor containing 120 thermocouples. Prior to analysis, each sample was sealed in an aluminum pan. Compound **1** was investigated between 193 and 343 K, whereas compound **2** was measured between 193 and 373 K, i.e., below their degradation point (compare Figures 1 with S5 and S6 in Supporting Information). The experimental protocol for compound **1** consisted of three stages: (I) an initial heating scan from 193 to 343 K at a rate of 10 K/min, (II) subsequent cooling run to 193 at −10 K/min, and (III) the final, second heating scan up to 343 at 10 K/min. In turn, the experimental protocol for compound **2** involved five stages: (I) an initial heating scan from 193 to 373 K at a rate of 10 K/min, (II) cooling run to 193 K at a rate of −10 K/min, (III) a second heating scan up to 373 at 10 K/min, (IV) a second cooling cycle to 193 at −15 K/min, and (V) a final heating scan to 373 at 10 K/min. All measurements were performed under a nitrogen atmosphere with a flow rate of 60 mL/min.

### 2.5 Broadband Dielectric Spectroscopy (BDS)

Broadband dielectric studies of compounds **1** and **2** were performed under ambient-pressure conditions by means of a Novocontrol Broadband Dielectric Spectrometer equipped with an Alpha Impedance analyzer. Measurements were performed in a stainless-steel parallel-plate capacitor with a 100 μm gap between the plates maintained by two quartz spacers. Dielectric spectra were recorded between 0.1 Hz and 1 MHz in the temperature range of 143–241 K (compound **1**) or 153–245 K (compound **2**), with data collected every 5 K below *T*_g_ and every 2 K near or above *T*_g_. Temperature control and temperature stabilization were ensured by a Novocontrol Quattro system and nitrogen gas. The accuracy of the temperature stabilization was set to ±0.1 K.

Prior to measurements, each compound was vitrified by first heating it above its melting point between the capacitor plates, followed by rapid quenching using a copper plate placed in liquid nitrogen. To prevent crystallization, the vitrified samples were quickly transferred (while still cold) into a precooled cryostat, where the temperature was immediately set to 143 K (compound **1**) or 153 K (compound **2**), ensuring the sample remained below its glass transition temperature. Throughout sample preparation, the capacitor plate temperatures were monitored using a thermocouple. Dielectric measurements were performed progressively from the lowest to the highest temperature. We also repeated some dielectric measurements for ester derivatives **3** and **4**. However, for these compounds the dielectric spectra were collected only at 143 and 233 K.

All of the collected complex dielectric permittivity spectra were analyzed using the WinFit software provided by Novocontrol. The structural α relaxation was modeled using a single Havriliak–Negami function

1 $$\varepsilon^{*}\left(\omega \right)=\varepsilon_{\infty }+\frac{\Delta \varepsilon }{{\left(1+{\left(i\omega \tau_{HN}\right)}^{\alpha_{HN}}\right)}^{\beta_{HN}}}$$

where, ε* represents the complex dielectric permittivity, ε_∞_ denotes its high-frequency limit, Δε corresponds to the dielectric strength, ω signifies the angular frequency, τ_HN_ refers to the Havriliak–Negami relaxation time, whereas α_HN_ and β_HN_ characterize the symmetric and asymmetric broadening of the relaxation process, respectively.^15^ The secondary β process was described with a Cole–Cole function

2 $$\varepsilon^{*}\left(\omega \right)=\varepsilon_{\infty }+\frac{\Delta \varepsilon }{1+{\left(i\omega \tau_{CC}\right)}^{\alpha_{HN}}}$$

where, τ_CC_ represents the Cole–Cole relaxation time, while the definitions of the remaining parameters remain consistent with those used in the Havriliak–Negami function.^16^ The corresponding relaxation times τ_α_ and τ_β_ were determined using the following equations^1^

3 $$\tau_{\alpha }=\tau_{HN}{\left[\sin\left(\frac{\alpha_{HN}\pi }{2\beta_{HN}+2}\right)\right]}^{-1/\alpha_{HN}}{\left[\sin\left(\frac{\alpha_{HN}\beta_{HN}\pi }{2\beta_{HN}+2}\right)\right]}^{1/\alpha_{HN}}$$

4 $$\tau_{\beta }=\tau_{CC}$$

### 2.6 Density Functional Theory (DFT) Calculations

DFT calculations^17,18^ were carried out using the hybrid B3PW91 functional^19^ combined with the 6-311++G­(d,p) basis set^20–22^ to explore the conformational diversity and possible conformational changes in compounds **1–5**. This computational approach was chosen due to its previously demonstrated accuracy in reproducing experimental energy barriers for intramolecular conformational transitions in structurally related molecules.^23,24^ The initial step of the analysis involved a full structure optimization of two selected conformers (S_1_ and S_5_) of both compounds **1** and **2**, as well as conformers A_1_, P_1_, and B_1_ for compounds **3**, **4**, and **5**, respectively. These calculations were performed with tight convergence criteria and a default grid. All the geometries mentioned above are summarized in Tables S1 and S2 in Supporting Information. In turn, the numbering scheme for non-hydrogen atoms in compounds **1**–**5**, used in the DFT calculations, is illustrated in Figure S9 and repeated in the Results and Discussion section for clarity.

A detailed conformational analysis in compounds **1** and **2** was performed for exemplary dihedral angles φ_2‑24‑27‑4_ and φ_18‑1‑5‑13_. This involved a stepwise alteration in their value with increments of ±5° using the ModRedundant scan approach while tracking the corresponding changes in energy and dipole moment magnitude. This exploration led to the identification of additional low-energy conformers, labeled S_2_–S_4_, which were subsequently reoptimized under the same tight convergence criteria and default grid settings. Conformational analysis was also performed for compounds **3–5** and involved a stepwise alteration in the dihedral angle φ_2‑22‑25‑29_ value, starting from the optimized geometries A_1_, P_1_, and B_1_. Additionally, compound **4** was examined for conformational transitions related to the dihedral angle φ_28‑30‑41‑42_, while for compound **5**, this analysis targeted the dihedral angles φ_28‑30‑37‑40_ and φ_30‑37‑40‑41_. All DFT calculations were conducted using the Gaussian 16 software package, Revision C.01.^25^

## 3 Results and Discussion

Compounds **1** and **2** were obtained as crystalline solids following synthesis, with the melting temperatures of approximately 315 and 343 K, respectively. The melting process is evident in the thermograms as a single endothermic peak (see the black curves in Figure 1b,c). Thermogravimetric analysis (TGA) confirms that melting occurs without any thermal degradation, as mass loss begins only above 410 K for both compounds (Figures S5 and S6 in the Supporting Information). The mass loss proceeds in a single-step process, with the maximum rate observed at approximately 505 K for compound **1** and 485 K for compound **2**. As revealed by calorimetry, the nondegraded melted compounds can be supercooled and vitrified when cooled rapidly. For compound **1**, a cooling rate of 10 K/min was sufficient to fully convert the supercooled liquid into a glass without cold crystallization. In contrast, compound **2** required a higher cooling rate of approximately 15 K/min to achieve vitrification. During the subsequent heating scan at 10 K/min, the glass transition is manifested as a weak thermal effect at *T*_g_ = 212 ± 1 K for compound **1** and *T*_g_ = 223 ± 1 K for compound **2** (see red curves in Figure 1b,c). This result indicates that the *T*_g_ value increases with molar mass among 1,2-bis­(2-halogenethylthio)-4-methylbenzenes, which is an opposite trend to that observed previously for the ester derivatives of 1,2-bis­(2-hydroxyethylthio)-4-methylbenzene (compounds **3–5**). In that series, *T*_g_ gradually decreases from 220 to 202 K as the acid unit in the ester group extends from the two-carbon acetyl to the four-carbon butyryl moiety, with measured values of 220 ± 1 K for compound **3**, 211 ± 1 K for compound **4**, and 202 ± 1 K for compound **5**.^11^ This findings highlight that flexible, linear chains with weak intermolecular interactions constitute one of the key factors influencing *T*_g_ in nonpolymeric molecular glass-formers, consistent with the recent studies employing RNN analysis.^10^

In contrast to compounds **3–5**, the substances **1** and **2** exhibit also a significant tendency toward cold crystallization upon reheating at 10 K/min from the supercooled liquid state, marked by a sharp exothermic peak on the thermograms. Under these conditions, the crystallization onset occurs at approximately 263 K for both compounds **1** and **2**, that is at temperatures higher by roughly 51 and 40 K than their respective *T*_g_ values. The higher cooling rate needed to prevent crystallization, along with the closer onset of recrystallization to *T*_g_, suggests that compound **2** is more prone to crystallization than compound **1**. Nevertheless, the common feature of both compounds is the apparent recrystallization into a different polymorphic form, as indicated by their reduced melting temperatures. Such behavior is characteristic of conformationally flexible molecules,^26–28^ a group to which compounds **1** and **2** belong. Unfortunately, attempts to grow single crystals suitable for X-ray diffraction were unsuccessful. Consequently, further investigations are required to fully elucidate the polymorphism of these materials.

Figure 2a,b shows representative dielectric loss spectra of compounds **1** and **2**, ε″(*f*), measured on the amorphized materials both above and below their glass transitions and presented as a function of frequency. Both compounds exhibit a similar dielectric response, with relaxation processes occurring in the glassy and liquid states. The glass phase of each compound is characterized by a single, broad, and symmetric relaxation of low intensity, with its maximum appearing only below 183 K within the frequency range of 10^–1^–10^6^ Hz. Consequently, we classify this process as a secondary relaxation. In contrast, the relaxation process apparent above *T*_g_ is characterized by an asymmetric and much narrower profile. Its amplitude is approximately 2 orders of magnitude higher than that of the β relaxation. This process is also followed exclusively by a conductivity branch. Therefore, it can be identified as the structural α relaxation originating from the cooperative motion of molecules in the liquid phase. The α-relaxation amplitude starts decreasing rapidly above 237 K in compound **1**, and 243 K in compound **2**. The decline is accompanied by a significant reduction in static dielectric permittivity, ε_s_′, indicating its association with cold crystallization from the supercooled liquid. Dielectric measurements further confirm that both compounds display a considerable tendency toward recrystallization, as the process begins once the α relaxation peak shifts beyond only *f*_c_ ≈ 1 kHz, corresponding to a relaxation time of $\tau_{c}=\frac{1}{2\pi f_{c}}\approx 160\;\mu s$ .

**Figure 2:**
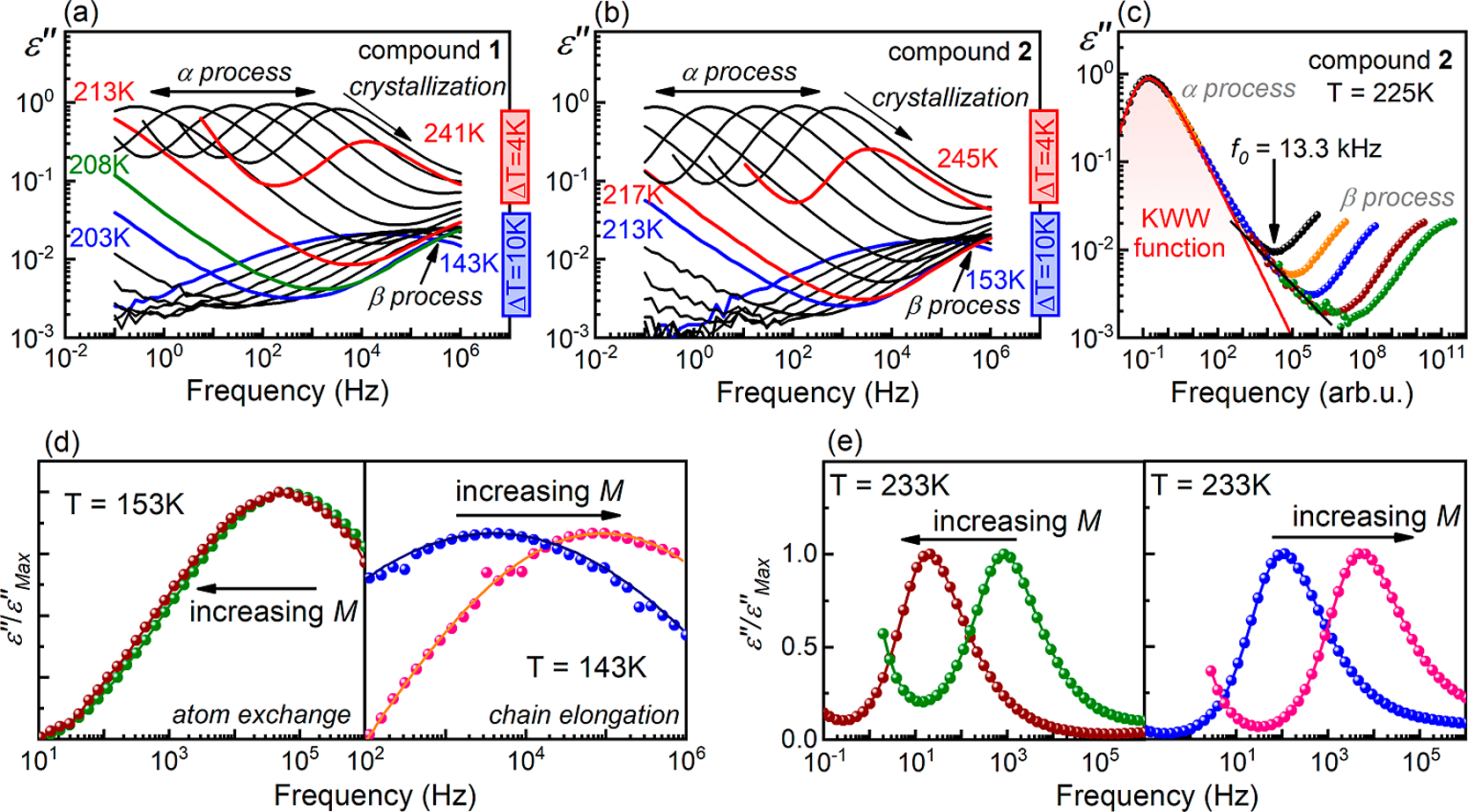
(a) Frequency-dependent ε″(*f*) spectra collected for compound **1** above and below its *T*_g_. (b) Selected ε″(*f*) spectra measured for compound **2** between 153 and 245 K. (c) Master plot constructed for compound **2** by aligning the α-loss peak captured at 225 K with the loss spectra recorded at lower temperatures. The red line represents the KWW fit function β_KWW_ = 0.58, while the black line highlights the excess wing. (d) Contrasting influence of increasing side chain molar mass on the position of the β-relaxation peak, resulting from either halogen substitution or chain extension. The ε″(*f*) spectra measured at 153 K and normalized to the maximum of losses are shown in green and red for compounds **1** and **2**, respectively, while those for the acetyl (compound **3**) and propionyl (compound **4**) ester derivatives at 143 K are depicted in blue and pink. (e) Divergent impact of increased side chain molar mass on the α-relaxation peak position, driven by halogen substitution or chain elongation. ε″(*f*) spectra of compounds **1**, **2** and their acetyl and propionyl ester analogues are color-coded in green, red, blue, and pink, respectively.

Apart from the α and β processes, the dielectric loss spectra of compounds **1** and **2** are characterized by an additional excess wing. This feature becomes more pronounced when transforming the ε″(*f*) into the temperature domain ε″(*T*), where it appears as a kink in the low-temperature flank of the α relaxation (see Figure S7a,b in Supporting Information). Its presence is also evident in ε″(*f*) after constructing a master plot, i.e., a curve obtained by aligning the α-loss peak clearly visible near *T*_g_ with loss spectra recorded at lower temperatures (where only the high-frequency flanks of the α-loss peak were measured) achieved by horizontally shifting them toward higher frequencies (cf., Figures 2c and S7c in Supporting Information). As a reference spectrum, we chose that measured at 217 K for compound **1** and 225 K for its bromine analogue **2**. The well-resolved α relaxation peak was additionally fitted to the one-side Fourier transform of the Kohlrausch–Williams–Watts (KWW) function

5 $$\varepsilon^{*}\left(\omega \right)=\Delta \varepsilon \int_{0}^{\infty }e^{i\omega t}\left[-\frac{d}{dt}\exp\left(-{\left(\frac{t}{\tau_{\alpha }}\right)}^{1-n}\right)\right]\;dt$$

where, ε* is the complex dielectric permittivity, Δε represents the dielectric strength, ω is the angular frequency, τ_α_ is the structural relaxation time under given temperature–pressure conditions, and *n* is the coupling parameter linked to the stretch exponent β_KWW_ of the KWW function by the relation *n* = 1 – β_KWW_.^29,30^ As depicted in Figures 2c and S7c, the KWW function provides a good fit to the α-loss peak near its maximum for both compounds. The best fits are obtained for τ_α_ = 0.59 s and β_KWW_ = 0.55 for compound **1** and τ_α_ = 0.96 s and β_KWW_ = 0.58 for compound **2**. The plausible origin of the excess wing was further explored using the Coupling Model, which establishes a correlation between the relaxation times of the α process (τ_α_) and the secondary Johari–Goldstein (JG) relaxation (τ_JG_), typically associated with the restricted reorientation of entire molecules.^31^ The relationship follows the equation

6 $$\tau_{JG}\left(T\right)≅\tau_{0}\left(T\right)=t_{c}^{n}\tau_{\alpha }{\left(T\right)}^{1-n}$$

where, τ_0_ is the ‘primitive’ relaxation time, and *t*_c_ is the crossover time marking the transition from independent to cooperative relaxation (1–2 ps).^32,33^ Using Formula [Disp-formula eq6] along with the extracted τ_α_ and β_KWW_ values, the τ_0_ parameter was calculated as 4 and ∼12 μs for compounds **1** and **2**, respectively. Accordingly, the expected frequencies *f*_0_ for detecting the Johari–Goldstein (JG) secondary relaxation are approximately 39.2 and 13.3 kHz in these compounds. In both analogues, these predicted values align well with the kink observed in the high-frequency slope of the α-loss peak, supporting its likely origin as a signature of the intermolecular JG process. In contrast, these values do not correspond to the observed β-relaxation peaks, reinforcing their classification as a non-JG relaxation associated with intramolecular conformational changes within the polar side chains of the aromatic ring. Such an image with a single intramolecular relaxation is consistent with findings from the esters of 1,2-bis­(2-hydroxyethylthio)-4-methylbenzene (compounds **3**–**5**), which are structural analogues of compounds **1** and **2**.^11^ However, our results indicate that in the ester series, the secondary relaxation process shifts toward higher frequencies at a constant temperature as the molar mass of the aromatic ring side chains increases, while compounds **1**, **2** exhibit the opposite trend, with the intramolecular secondary relaxation shifting only slightly toward lower frequencies under the same conditions (see Figure 2d). A similar pattern emerges in the α-relaxation process (Figure 2e). Among the 1,2-bis­(2-hydroxyethylthio)-4-methylbenzene esters, increasing the molar mass of the aromatic ring side chains causes the α-loss peak to shift toward higher frequencies. However, in the halogenated derivatives **1** and **2**, the α-relaxation instead moves toward lower frequencies, highlighting a fundamental difference in the molecular dynamics between these two groups of compounds.

To gain deeper insight into this issue, we analyzed the dielectric spectra of compounds **1** and **2**, extracting relaxation times under varying temperature conditions according to the procedures described in Section 2.5. As shown in Figure 3a,b, the selected fitting approach accurately captures the experimental data, particularly around the relaxation peak maximum. Figure 3c illustrates the determined values of the structural and secondary relaxation times, τ_α_ and τ_β_, as a function of the temperature inverse. As presented, τ_α_ of compounds **1** and **2** evolves in a super-Arrhenius fashion near *T*_g_, whereas τ_β_ follows an Arrhenius-like pattern. This behavior closely aligns with the relaxation dynamics previously observed in esters of 1,2-bis­(2-hydroxyethylthio)-4-methylbenzene (see Figure S8).^11^ Therefore, we fit the temperature dependences of τ_α_ and τ_β_ with the Vogel–Fulcher–Tamman eq 7 and the Arrhenius law eq 8, respectively

7 $$\tau_{\alpha }\left(T\right)=A\exp\left(\frac{B}{T-T_{0}}\right)$$

8 $$\tau_{\beta }\left(T\right)=\tau_{0}\left(\frac{E_{a}}{RT}\right)$$

In these equations, *A* is a pre-exponential factor, *B* is a material constant, *T*_0_ signifies the ideal glass temperature, *R* denotes the gas constant, τ_0_ refers to the β relaxation time at the limit of an infinitely high temperature, and *E*_a_ is the activation energy of the β process.^34–36^ The last parameter can be interpreted as the average energy barrier associated with conformational changes within the molecular skeleton, which alters the amplitude and/or orientation of the dipole moment vector.

**Figure 3:**
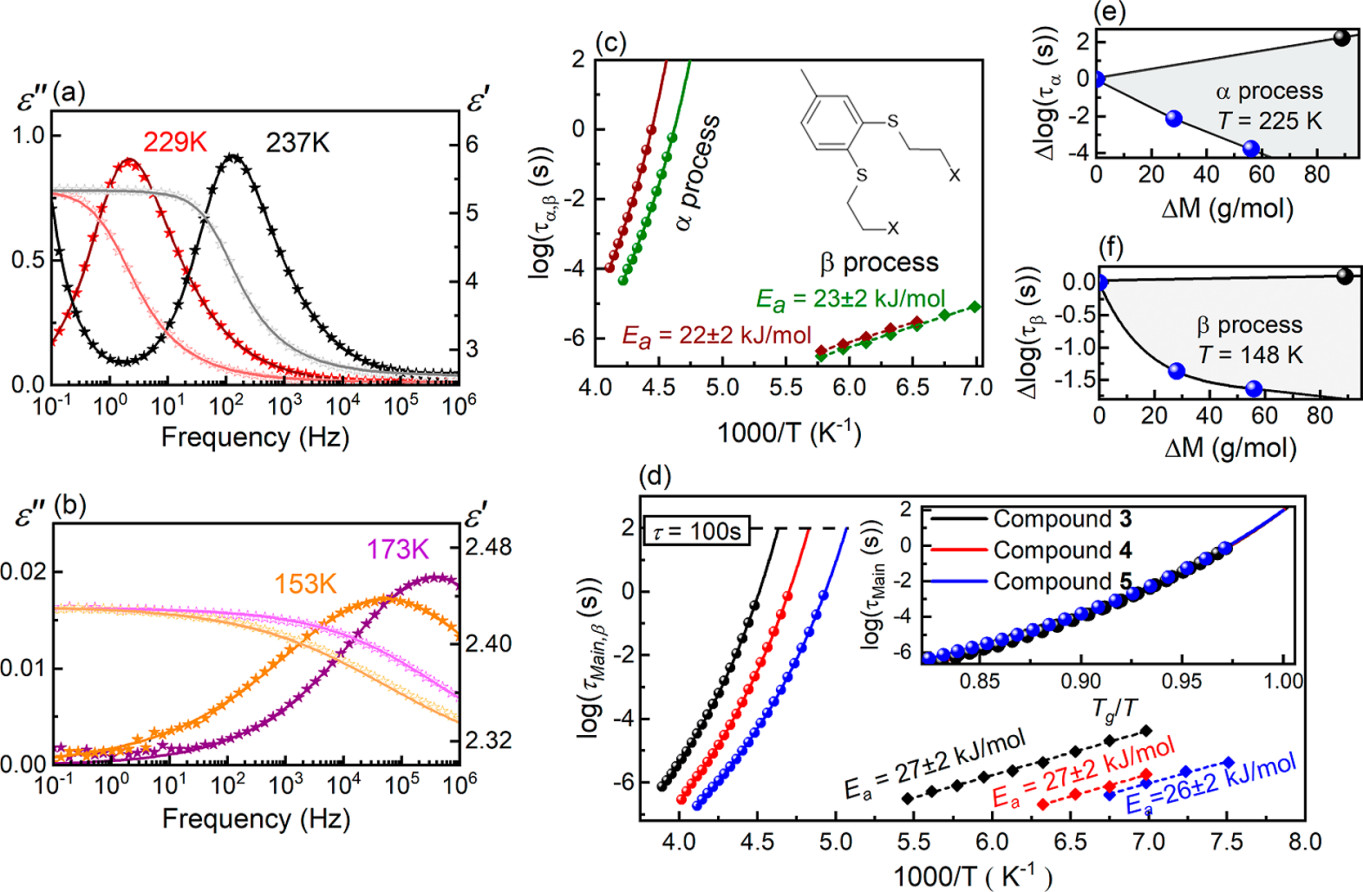
(a) The Havriliak–Negami fit functions superimposed on representative ε′(*f*) (open symbols) and ε″(*f*) spectra (closed symbols) of compound **2** collected at 229 and 237 K. (b) ε′(*f*) (open symbols) and ε″(*f*) spectra (closed symbols) of compound **2** measured at 153 and 173 K and fitted using the Cole–Cole formalism. (c) Temperature evolution of relaxation times τ_α_ and τ_β_ for compounds **1** (green symbols) and **2** (red symbols). (d) Temperature dependence of relaxation times τ_α_ and τ_β_ for compounds **3** (black symbols), **4** (red symbols), and **5** (blue symbols). The inset highlights the similarity in τ_α_(*T*) for compounds **3**–**4**. Reprinted from ref 11, with permission from Elsevier. (e) Changes in τ_α_ caused by increasing molar mass of the aromatic ring side chains achieved by the halogen atom exchange (black dots) and chain elongation (blue dots). (f) Changes in τ_β_ resulting from increased molar mass of the aromatic ring side chains via halogen atom exchange (black dots) and chain elongation (blue dots).

The optimal parametrization of the experimental τ_α_(*T*) curves is obtained for log *A*, *B*, and *T*_0_ parameters equal to −13.73 ± 0.36, 1420 ± 90 K, 171 ± 2 K for compound **1**, and −14.31 ± 0.36, 1550 ± 90 K, 178 ± 2 K for compound **2**, respectively. According to these fitting parameters, τ_α_ reaches 100 s (a characteristic time scale of the structural relaxation commonly associated with the glass transition in low-molecular-weight glass-formers^1^) at approximately 211 ± 1 K for compound **1** and 220 ± 1 K for compound **2**. These temperatures agree with the calorimetric *T*_g_ values, supporting our previous assignment of the main α process to structural relaxation associated with cooperative molecular reorientations.

In contrast to τ_α_, the τ_β_(*T*) is accurately described by the Arrhenius law with log τ_0_ and *E*_a_ values of −13.38 ± 0.14, 23 ± 2 kJ/mol for compound **1**, and −12.85 ± 0.15, 22 ± 2 kJ/mol for compound **2**. Notably, the ratio *E*_a_/*RT*_g_ (with *T*_g_ determined by extrapolating the VFT fit to the temperature at which τ_α_ = 100 s) is equal to approximately 13.1 and 12.1 for the β relaxations of compounds **1** and **2**, respectively. These values remain consistently below 24. According to the concept of Kudlik et al., such secondary relaxations that deviate from the empirical rule ⟨*E*_a_⟩ = 24*RT*_g_ are considered intramolecular processes.^37–40^ Consequently, it is likely that the β relaxation in compounds **1** and **2** originates from conformational changes within the side chains of the aromatic ring, as previously indicated for the analogous esters of 1,2-bis­(2-hydroxyethylthio)-4-methylbenzene.^11^ This interpretation, based on the *E*_a_/*RT*_g_ ratio, aligns with our prior predictions using the Coupling Model.

Despite this similarity, a comparison of Figure 3c,d highlights a distinct difference between the halogen and ester derivatives of 1,2-bis­(2-hydroxyethylthio)-4-methylbenzene. In the case of halogen derivatives, increasing the molar mass by substituting chlorine atoms with bromines leads to an increase in structural relaxation time τ_α_ by approximately 2 decades at a given temperature (Figure 3c). In contrast, a similar effect of molar mass rise achieved by the elongation of the aromatic ring side chains (as seen among the ester derivatives) induces the opposite effect, shortening the τ_α_ (Figure 3d).

This contrasting influence of the molar mass is particularly evident near *T*_g_, as shown in Figure 3e. For instance, at 225 K, replacing chlorine with bromine increases the molar mass by 89 g/mol and simultaneously extends τ_α_ by two decades. Meanwhile, increasing the ester group size from acetyl to butyryl accelerates structural relaxation by four decades. Notably, the influence of the ester group length on relaxation dynamics follows a nonlinear trend, which becomes even more pronounced in the case of intramolecular secondary relaxations (Figure 3f). As depicted for the exemplary temperature conditions of 148 K, the most significant acceleration of β relaxation, approximately 1.5 decades, occurs when transitioning from an acetyl to a propionyl group. Further increasing the molar mass by the same value (28 g/mol) achieved by substituting propionyl with a butyryl group results in a much smaller effect, i.e., shortening the β-relaxation time τ_β_ by only 0.5 decades. Notably, increase in the molar mass by 89 g/mol due to simple halogen atom change from chlorine to bromine slightly decelerate the intramolecular molecular dynamics. The impact is, however, very small.

Further DFT calculations were conducted to investigate the origin of the comparable activation energies and relaxation times associated with the secondary intramolecular β relaxation observed in compounds **1** and **2**. As shown in Figures 4a and S10, both molecules exhibit multiple stable conformations that differ in their dipole moment magnitude. Notably, despite the substantial difference in the size of the halogen substituents, both molecules can adopt analogous conformations. Representative conformers (S_1_–S_5_) for each compound are illustrated in Figures 4a and S10, with detailed structural data summarized in Table S1. The results in Table S1 indicate that replacing chlorine with bromine introduces only slight changes in the dihedral angles within the side chains attached to the aromatic ring.

**Figure 4:**
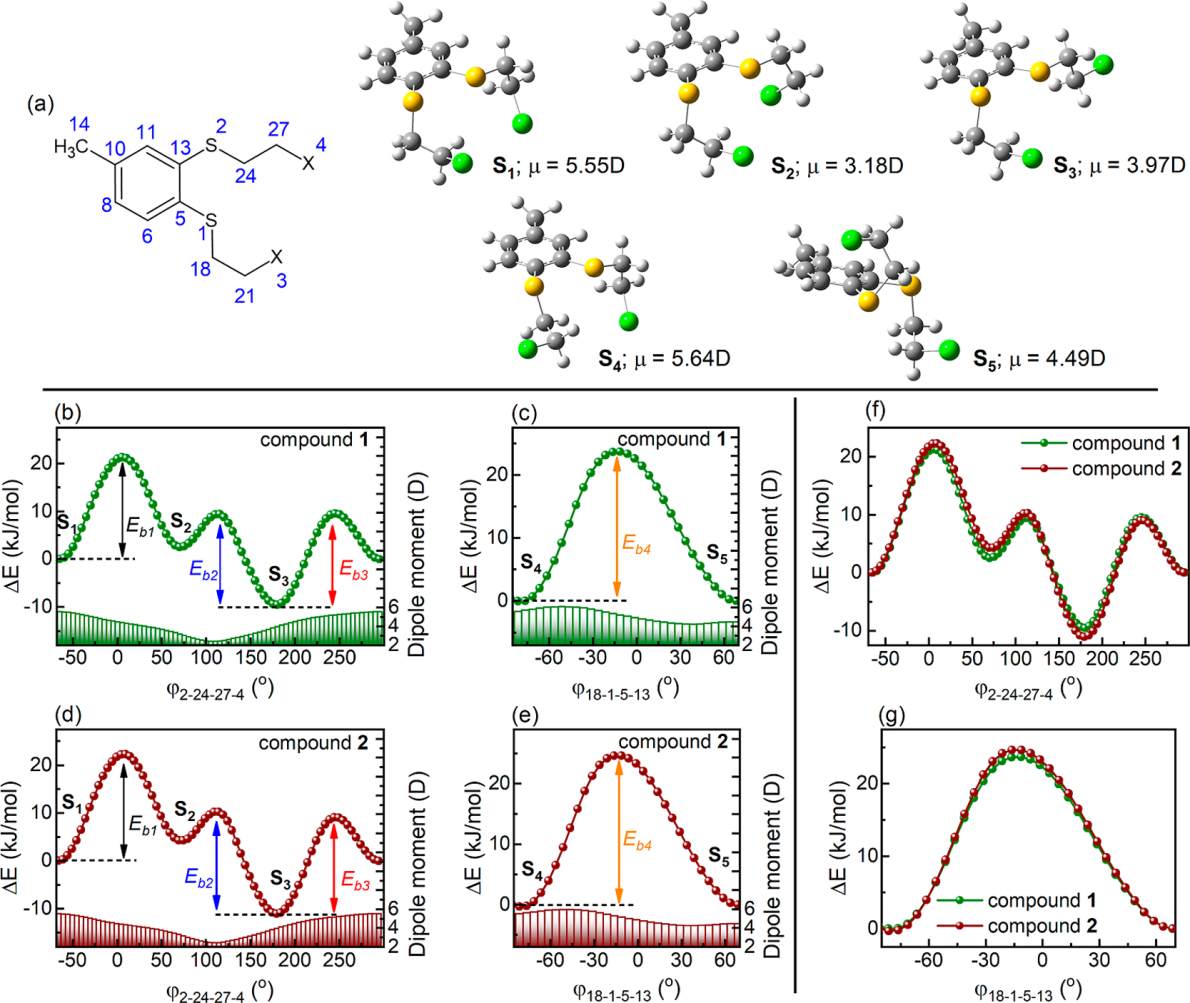
(a) Chemical structure of compounds **1** and **2** with the adopted numbering scheme of non-hydrogen atoms. The DFT-optimized representative conformers S_1_–S_5_ of compound **1** presented the corresponding dipole moment values. (b) Changes in potential energy (points) and dipole moment (bars) of compound **1** achieved by the alteration in its dihedral angle φ_2‑24‑27‑4_, when structure S_1_ is used as an initial geometry. (c) Conformational transition between S_4_ and S_5_ in compound **1**, driven by rotation around φ_18‑1‑5‑13_, with corresponding changes in potential energy and dipole moment. (d) Changes in potential energy and dipole moment as a function of the dihedral angle φ_2‑24‑27‑4_ in compound **2**, starting from geometry S_1_. (e) Conformational switching between S_4_ and S_5_ in compound **2**, induced by rotation around φ_18‑1‑5‑13_, with the associated energy and dipole moment variations. (f) Comparison of energy profiles for compounds **1** and **2**, highlighting similar rotational effects for the dihedral angle φ_2‑24‑27‑4_. (g) Overlay of energy profiles for compounds **1** and **2**, illustrating similar characteristics at φ_18‑1‑5‑13_.

Likewise, changes in energy and dipole moment associated with conformational transitions remain comparable between the two compounds. One notable example is the transformation of conformer S_1_ into S_2_, driven by a gradual increase in the dihedral angle φ_2‑24‑27‑4_ from −65° to 70°, as shown in Figure 4b,d. This process proceeds with energy barriers of 21.3 kJ/mol for compound **1** and 22.3 kJ/mol for compound **2**, closely matching the experimentally determined activation energy of β relaxation in both substances. Interestingly, similar energy barriers are observed for the transitions S_3_ → S_2_ and S_3_ → S_1_, equal to 19.0 and 19.1 kJ/mol for compound **1** and 21.3 and 20.1 kJ/mol for compound **2**, respectively (see Figure 4b,d). Likewise, the mutual interconversion between conformers S_4_ and S_5_, driven by modifications in the dihedral angle φ_18‑1‑5‑13_, exhibits a comparable trend, with energy barriers of 23.7 kJ/mol for compound **1** and 24.6 kJ/mol for compound **2** (see Figure 4c,e). Each of these transitions is accompanied by notable changes in the dipole moment magnitude (as marked by bars in Figure 4b–e). Consequently, it is hard to distinguish which interconversion path may constitute the exact source of the β process in both compounds. It is even plausible that multiple interconversion routes collectively contribute to this relaxation process, depending on the molecular environment and local arrangement of neighboring molecules. This is consistent with the broad, symmetric shape of the β-relaxation peak, which conforms to the Cole–Cole formalism and indicates a distribution of relaxation times and activation energies.^41^ Nevertheless, the good agreement between the computed energy barriers and the experimentally determined activation energies supports the previous assignment of β relaxation as an intramolecular relaxation process associated with conformational changes, as previously suggested by the Coupling Model.

The DFT calculations also demonstrate that the overall energy profiles and changes in the dipole moment during conformational transformations are nearly identical for both compounds (see Figure 4f,g). Only minor discrepancies in the energy barriers are apparent, which generally do not exceed 1.5 kJ/mol. Such differences fall within the experimental uncertainty associated with the activation energy determination for the intramolecular dielectric β process. Consequently, these computational insights offer a rational explanation for the experimentally observed similarities in β-relaxation behavior between the two compounds, including their comparable relaxation times, activation energies, and relaxation magnitudes. Namely, the similarity in β-relaxation times τ_β_ between compounds **1** and **2** can be attributed to their nearly identical intermolecular interaction profiles and conformational transitions with similar energy barriers that proceed solely within the polar parts of the benzene ring side chains.

Conversely, extending the benzene ring side chains (as in the case of ester derivatives **3–5** of 1,2-bis­(2-hydroxyethylthio)-4-methylbenzene) enhances the molecular flexibility and conformational diversity. Each additional –CH_2_– unit triples the number of accessible conformers, broadening the range of molecular motions, including end-group, subgroup, and crankshaft movements.^12^ In esters **3–5**, these conformational changes can occur in both the polar and nonpolar regions of the benzene ring side chains, offering a clear explanation for the observed differences in τ_β_ values despite identical activation energies. Namely, the comparable *E*_a_ values (26–27 kJ/mol) suggest a common underlying relaxation mechanism involving conformational changes in the polar segments of the benzene ring side chains. In turn, the progressive shortening of τ_β_ with increasing side-chain length reflects an enhanced ease of spatial rearrangements, stemming from enhanced molecular flexibility introduced by the elongation of the nonpolar regions. This interpretation is supported by DFT calculations, which reveal that some conformational changes in the polar domains of compounds **3–5** (such as a dihedral angle φ_2‑22‑25‑29_ alteration) involve energy barriers close to the experimentally determined *E*_a_ values of β relaxations (see Figure 5a). These transitions encompass the ester −COO– group reorientations and result in significant changes in dipole moment magnitude, making them dielectrically active in all three esters. In contrast, conformational changes in the nonpolar regions are linked to much lower energy barriers and cause only minor fluctuations in the dipole moment. A clear example is the alteration in dihedral angle φ_28‑30‑41‑42_ in compound **4**, or the changes in dihedral angles φ_28‑30‑37‑40_ and φ_30‑37‑40-41_ in compound **5**, all of which are associated with energy barriers of only a few kJ/mol (see Figure 5b–d). If such low-barrier transitions are dielectrically active, they would give rise to much faster secondary relaxations within a single compound than the higher-energy β processes—too rapid to be detected in esters **3**–**5** between 0.1 Hz and 1 MHz. Nevertheless, the increased flexibility and additional dynamic changes within the nonpolar end groups may facilitate dielectrically active conformational rearrangements elsewhere in the side chains, thereby shortening τ_β_. This interpretation aligns with previous molecular dynamics simulations on esters **3** and **5**, which demonstrated that the overall orientational relaxation time of the side chains decreases with increasing side-chain length, even though the relaxation mechanism remains unchanged.^11^ It is also consistent with previous studies on other nonpolymeric systems, which reported no simple correlation between the relaxation times and activation energies of secondary relaxations.^42^ In fact, that research performed on the series of butane derivatives, aromatic ketones, saccharides or compounds with aromatic halobenzene rings showed that secondary relaxation times can depend not only on the relaxation mechanism but also on molar mass of the reorienting moiety, steric hindrance, or cross-conjugation effects.^42^ In line with that review, our results support the notion that while similar activation energies reflect common transition mechanisms, τ_β_ values are more sensitive to other molecular descriptors (such as flexibility). Halogen substitution in compounds **1** and **2** preserves the relaxation mechanism, rigidity, intermolecular interaction profile, and conformational landscape, ultimately resulting in almost overlapping τ_β_. In contrast, extending the nonpolar aliphatic tails increases internal flexibility, allowing for easier conformational rearrangements without modifying the relaxation mechanism, consequently shortening τ_β_ without alteration of *E*_a_.

**Figure 5:**
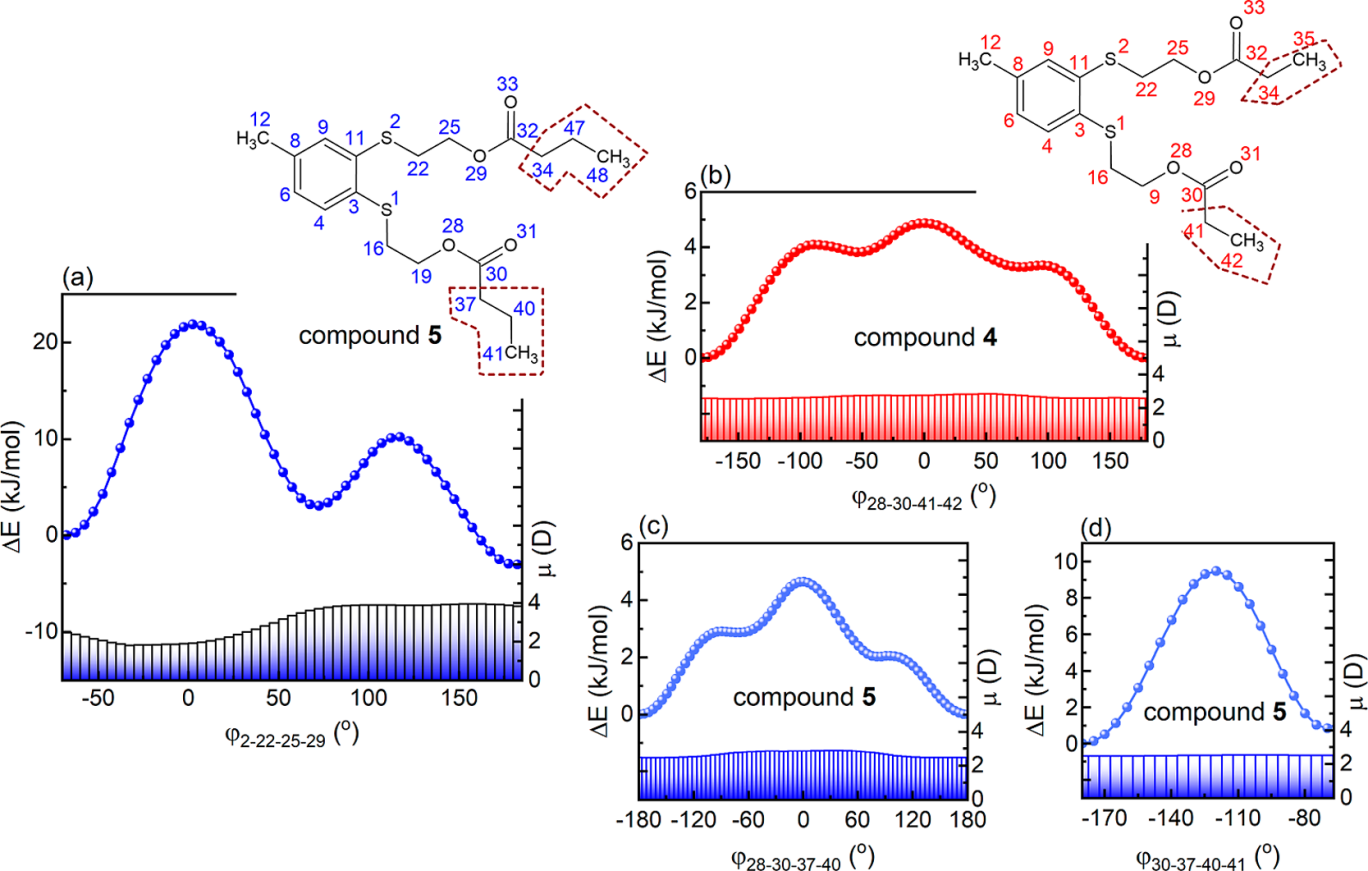
(a) Changes in potential energy (points) and dipole moment (bars) of ester **5** achieved by the alteration in its dihedral angle φ_2‑22‑25‑29_, when structure B_1_ is used as an initial geometry. The upper inset shows the chemical structure of compound **5** with the adopted numbering scheme of non-hydrogen atoms and marked nonpolar regions of the benzene ring side chains. (b) Changes in potential energy (points) and dipole moment (bars) of the ester **4** driven by rotation around its dihedral angle φ_28‑30‑41‑42_ when conformer P_1_ is used as the initial structure. The upper inset presents the molecular structure of compound **4**, including the numbering scheme applied to non-hydrogen atoms and marked the nonpolar segments of the benzene ring side chains. (c) Changes in potential energy and dipole moment as a function of the dihedral angle φ_28‑30‑37‑40_ in compound **5**, starting from geometry B_1_. (d) Low-energy-barrier conformational transition of structure B_1_ achieved by increasing the dihedral angle φ_30‑37‑40‑41_ value.

As a result, in halogen derivatives **1** and **2**, molar mass emerges as a key factor controlling the structural α relaxation times. The greater the molar mass, the more thermal energy (and the higher temperature) is required to induce molecular motion at a given rate. This effect is particularly pronounced in viscosity-related α-relaxation dynamics, as presented in Figure 3c,d. Thus, increasing molar mass through atomic substitution (such as replacing chlorine with bromine) decelerates molecular dynamics in the liquid phase and raises *T*_g_ according to the typical dependence *T*_g_ ∼ *M* ^α^.

Conversely, extending the benzene ring side chains (as in the case of ester derivatives **3–5** of 1,2-bis­(2-hydroxyethylthio)-4-methylbenzene) enhances molecular flexibility and conformational diversity, accelerating both inter- and intramolecular dynamics (see Figure 3e,f). Each additional –CH_2_– unit triples the number of accessible conformers, broadening the range of molecular motions.^12^ As demonstrated by previous molecular dynamics simulations, this disrupts efficient intermolecular packing, expands free volume, and ultimately facilitates molecular mobility.^11^ Consequently, greater intramolecular flexibility promotes easier molecular rearrangements in the liquid phase, reducing viscosity-related structural relaxation times at a given temperature and decreasing the *T*_g_ value.

These findings reveal that increasing molar mass can induce two opposing effects on relaxation dynamics and the glass transition temperature of nonpolymeric glass formers, depending on the accompanying changes in the internal molecular structure. Beyond molar mass, internal molecular flexibility and conformational diversity have emerged as key factors governing *T*_g_. This conclusion aligns with earlier observations that phase transition temperatures progressively decrease with increasing flexibility of functional groups covalently attached to a rigid molecular core. For example, a flexibility-induced decline in *T*_g_ (despite increasing molar mass) was reported in glass-forming phenothiazines and in derivatives of ibuprofen and naproxen.^43–45^ Moreover, a flexibility-driven decrease in the nematic–isotropic transition temperature was observed in liquid-crystalline α-(4-cyanobiphenyl-4′-yloxy)-ω-(1-pyrenimine-benzylidene-4′-oxy) alkanes.^46^ Setting aside the odd–even effect, a comparable trend was reported for α-4-[4-*n*-alkoxybenzoyloxy]-β-4‴-*N*,*N*-dimethylethylenes, where additionally longer alkoxy substituents lower the smectic–nematic transition temperature.^47^ These examples collectively underscore the critical role of internal molecular flexibility in modulating phase transition temperatures.

## 4 Conclusions

In this study, the complex relationship between the glass transition temperature and molecular structure was comprehensively analyzed by comparing the thermal and dynamic properties of structurally related 1,2-bis­(2-halogenethylthio)-4-methylbenzene and ester derivatives of 1,2-bis­(2-hydroxyethylthio)-4-methylbenzene.

Among them, 1,2-bis­(2-chloroethylthio)-4-methylbenzene (compound **1**) and 1,2-bis­(2-bromoethylthio)-4-methylbenzene (compound **2**) are two novel low-molecular-weight nonpolymeric halogen derivatives, exhibiting glass-forming properties with the *T*_g_ value of 212 ± 1 K and 223 ± 1 K, respectively. Both compounds show a pronounced tendency toward crystallization from the supercooled liquid state with the onset occurring at approximately 263 K. Their dielectric response is dominated by two well-resolved relaxation processes, observed both above and below *T*_g_. In the glassy phase, each compound exhibits a single secondary β relaxation, appearing as broad and symmetric loss peaks of low intensity in the imaginary part of the complex dielectric permittivity. This process has intramolecular nature and arises from conformational changes in the side-chains of the aromatic ring. Its relaxation times follow the Arrhenius law with the temperature-independent activation energy of approximately 22–23 kJ/mol for both compounds. In contrast, their structural α relaxation, apparent above *T*_g_ , resulting from the cooperative motion of entire molecules in the liquid, exhibits the super-Arrhenius character and follows the Vogel–Fulcher–Tamman equation close to the glass transition. This dielectric response is comparable to the one observed among the acetyl, propionyl, and butyryl ester derivates of 1,2-bis­(2-hydroxyethylthio)-4-methylbenzene. However, both groups of compounds exhibit a contrasting impact of growing molar mass on their overall molecular dynamics and *T*_g_ value.

When molar mass increases due to simple atomic substitution (e.g., replacing chlorine with bromine), *T*_g_ increases, and molecular mobility slows down at a given temperature as more thermal energy is required to induce motion of heavier molecules. This behavior arises also from nearly identical conformational transformations in structurally related compounds (such as the herein studied compounds **1** and **2**), where comparable energy barriers and intramolecular dynamics render molar mass the primary determinant of structural relaxation times. In contrast, a similar increase in molar mass achieved by elongating the flexible substituent attached to a rigid core (as observed among the acetyl, propionyl, and butyryl ester derivates) lowers the *T*_g_ value and enhances both the intra- and intermolecular dynamics. This is because extending the flexible moieties increases the number of accessible conformers and broadens the range of molecular motions, consequently, expanding free volume, facilitating molecular mobility, and ultimately shortening both the secondary and structural relaxation times. These findings reveal that internal molecular flexibility and conformational diversity are (beyond molar mass) crucial factors governing *T*_g_ and near-glass transition relaxation dynamics.

## Supplementary Material



## Notes

Chemical synthesis of compounds **1** and **2**; TGA and NMR data; additional information for dielectric data analysis; and details about the DFT-optimized geometries (PDF)
