# Supplementary material for: A Fresh Perspective on the Internal Plasticizing Effect in Non-Polymeric Glass-Formers
Source: J Phys Chem B. 2025 May 30;129(23):5831–41. doi: 10.1021/acs.jpcb.5c01809 (PMC12169652; doi:10.1021/acs.jpcb.5c01809)
Supplement: Supplementary file 1 [file jp5c01809_si_001.pdf]

A Fresh Perspective on the Internal Plasticizing Effect in Non-Polymeric Glass-Formers

Supplementary Information

Andrzej Nowok[a,*], Hubert Hellwig[b], Piotr Kuś[c]

[a]*Department of Experimental Physics, Wrocław University of Science and Technology, Wybrzeże Wyspiańskiego 27, 50-370 Wrocław, Poland*

[b]*Center for Integrated Technology and Organic Synthesis (CiTOS), MolSys Research Unit, University of Liège, B6a, Room 3/19, Allée du Six Août 13, 4000 Liège, Sart Tilman, Belgium*

[c]*Institute of Chemistry, University of Silesia, Szkolna 9, 40-006 Katowice*

[*]*andrzej.nowok@pwr.edu.pl*

1. Synthesis procedures

1.1. 1,2-bis(2-chloroethylthio)-4-methylbenzene

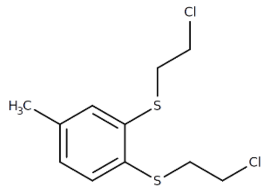

A solution of 1.02 g (4.2 mmol) of 1,2-bis(2-hydroxyethylthio)-4-methylbenzene in 10 mL of toluene was prepared in a 100 mL round-bottom flask equipped with magnetic stirrer and reflux condenser. The mixture was cooled in an ice bath to 0 °C and 1.6 mL (20 mmol) of $SOCl_2$ was added dropwise via syringe, after which the mixture was heated to reflux for 30 minutes. The mixture was allowed to stir over night at room temperature. Volatiles were distilled off and the crude product was purified by column chromatography (silica gel, hexane-CH_2Cl_2, 1:1 vol.). Yield: 0.76 g (65%) of a solid material. ^{1}H NMR (DMSO-d_6); δ (ppm): 7.34 (d, J = 7.9 Hz, 1H), 7.24 (s, 1H), 7.06 (d, J = 7.9 Hz, 1H), 3.77 (t, J = 7.3 Hz, 2H), 3.71 (t, J = 7.3 Hz, 2H), 3.30 (dt, J = 29.0, 7.3 Hz, 4H), 2.30 (s, 3H). ^{13}C NMR (DMSO-d_6); δ (ppm): 137.93, 136.60, 131.21, 131.13, 129.70, 128.20, 43.27, 43.25, 35.54, 34.82, 21.04. IR ν_{max}/cm^{-1} : 3073, 3050, 2964, 2921, 2853, 2731, 1892, 1583, 1544, 1503, 1456, 1439, 1422, 1383, 1292, 1261, 1211, 1119, 1039, 971, 868, 852, 805, 767, 752, 730, 698, 680, 633, 552, 447

1.2. 1,2-bis(2-bromoethylthio)-4-methylbenzene:

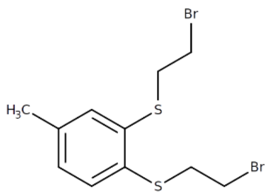

A solution of 7.40 g 1,2-bis(2-hydroxyethylthio)-4-methylbenzene (30.3 mmol) in 40 mL of CH_2Cl_2 was prepared in a two-neck 100 mL round-bottom flask equipped with magnetic stirrer and septum. The mixture was cooled in an ice bath to 0 °C and 2 mL (5.7 g, 21 mmol) of PBr_3 was added dropwise via syringe within 10 minutes. The mixture was stirred

for 6 h at room temperature, then 20 mL of ice-cold water was added, followed by stirring for an additional 15 minutes. Phases were separated. The organic phase was washed with water and dried with $MgSO_4$. Crude product was purified by column chromatography (silica gel, CH_2Cl_2:hexane, 4:1 vol.). Yield: 8.07 g (72%) of viscous liquid which solidifies after cooling to room temperature. ^{1}H NMR ($CDCl_3$); δ (ppm): 7.29 (d, J = 7.8 Hz, 1H), 7.16 (d, J = 1.9 Hz, 1H), 7.04 (dd, J = 7.8, 1.8 Hz, 1H), 3.55 – 3.45 (m, 4H), 3.37 – 3.25 (m, 4H), 2.36 (s, 3H). ^{13}C NMR ($CDCl_3$); δ (ppm): 138.34, 136.64, 131.74, 131.65, 130.52, 128.39, 35.83, 35.20, 29.79, 29.57, 21.15. IR ν_{max}/cm^{-1} : 3071, 3046, 3026, 2966, 2920, 2859, 1887, 1583, 1544, 1455, 1421, 1380, 1276, 1260, 1212, 1193, 1106, 1036, 944, 868, 849, 803, 740, 682, 602, 550, 444.

2. NMR spectra

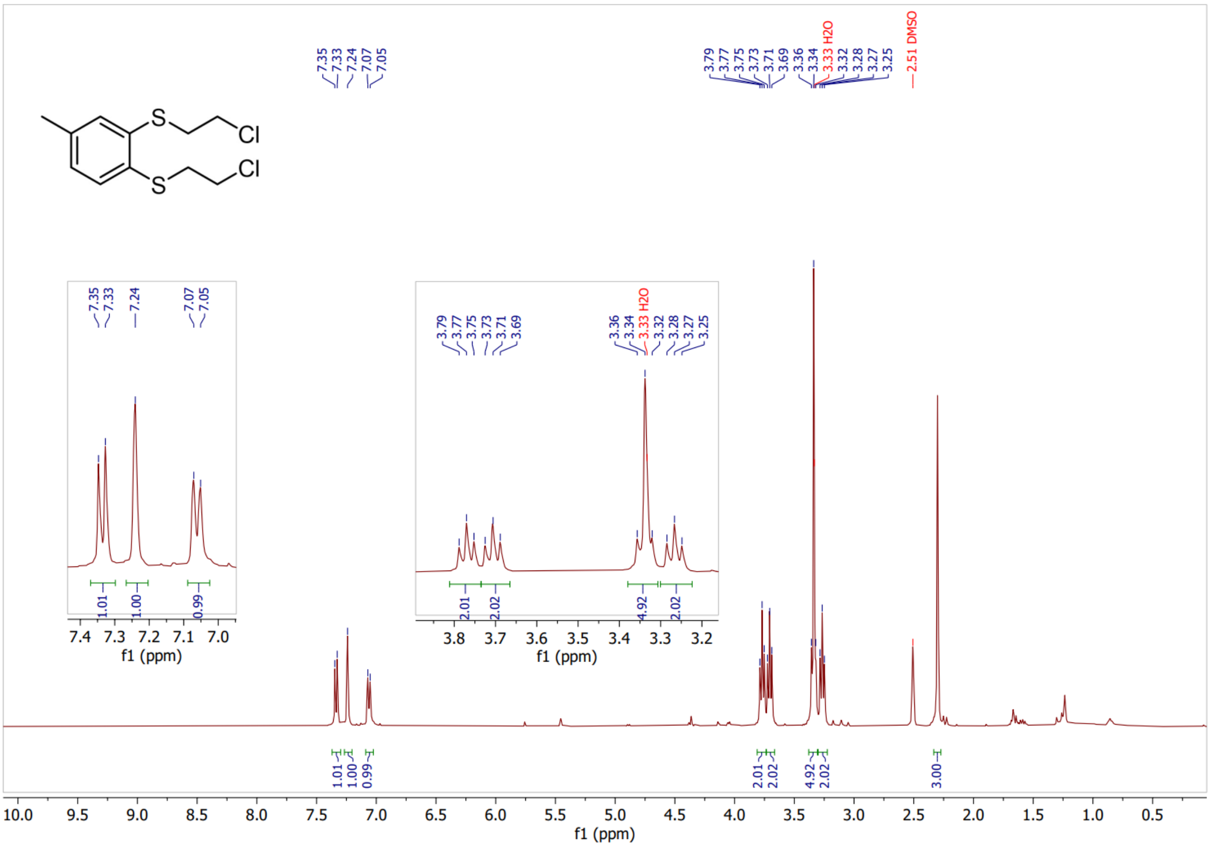

Figure S1. ^{1}H NMR spectrum of 1,2-bis(2-chloroethylthio)-4-methylbenzene in DMSO-d_6.

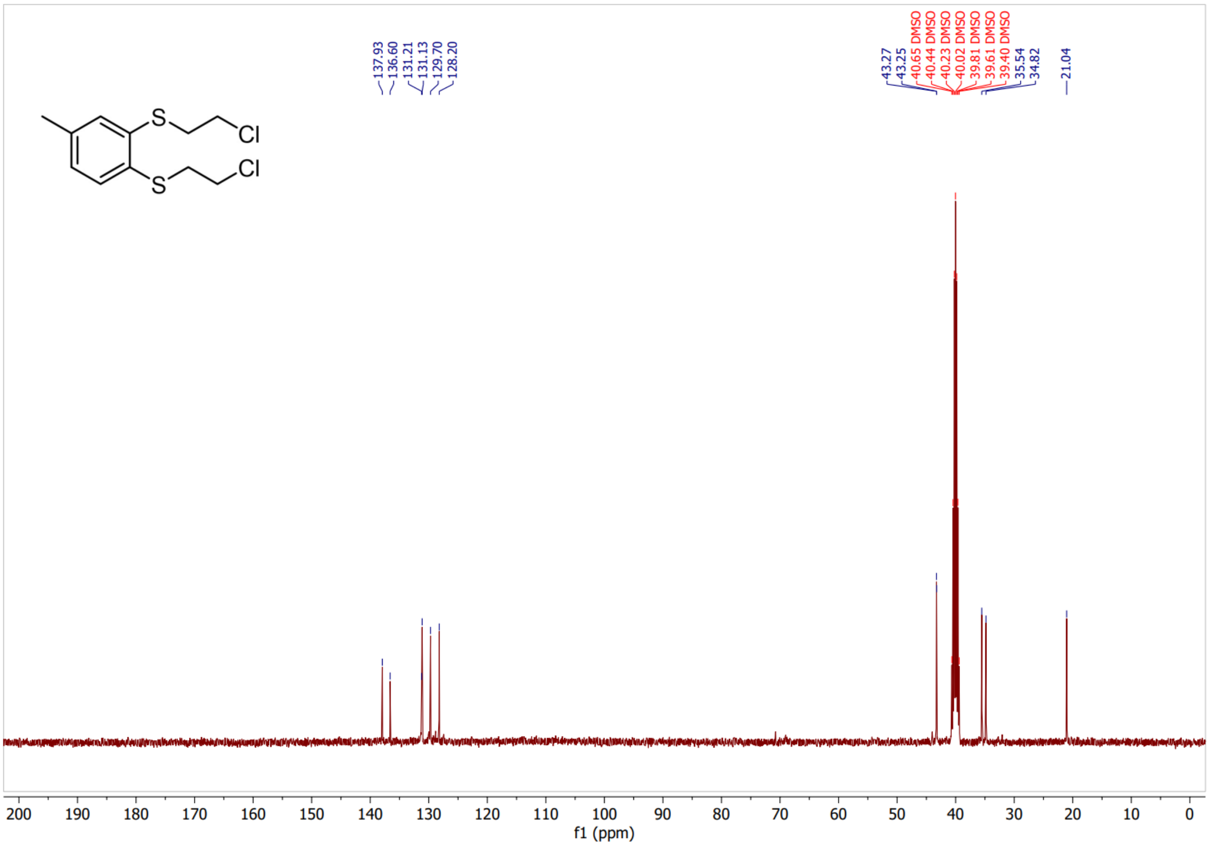

Figure S2. ^{13}C NMR spectrum of 1,2-bis(2-chloroethylthio)-4-methylbenzene in DMSO-d_6.

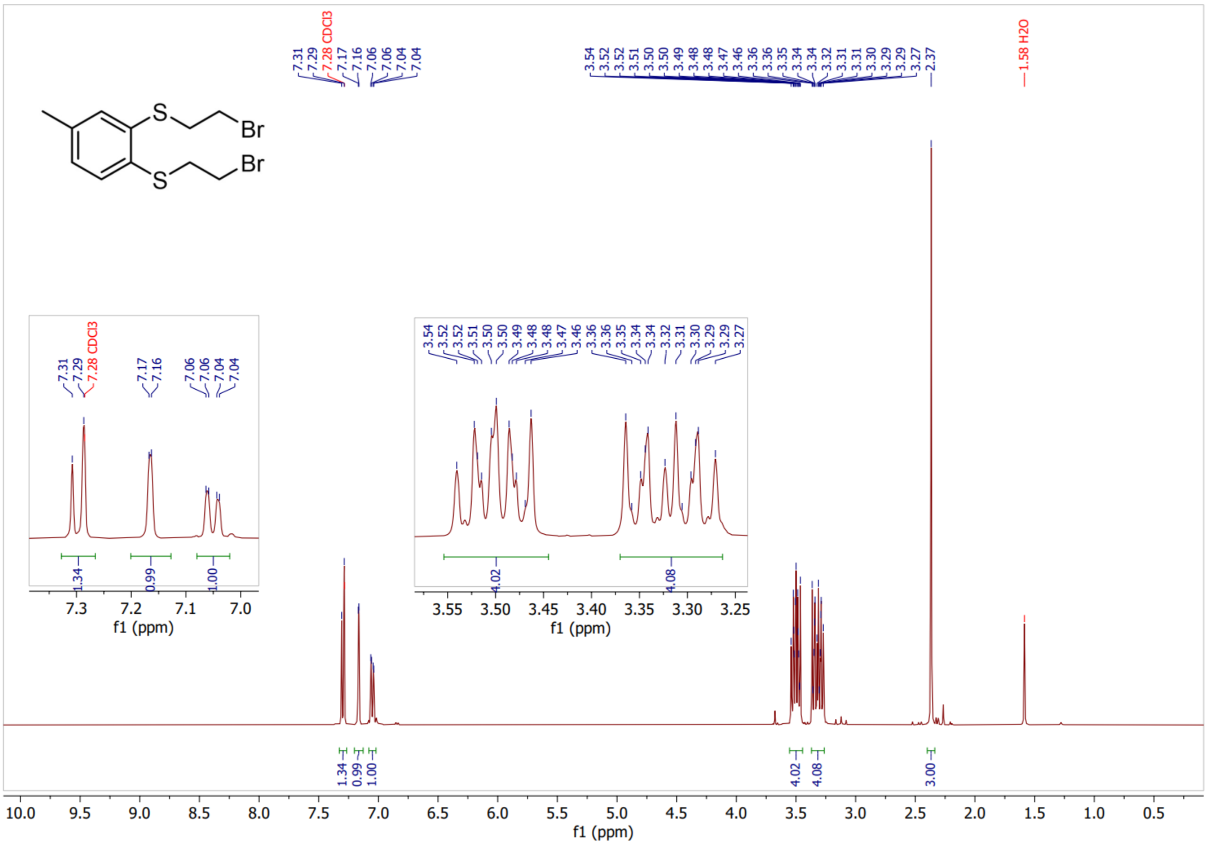

Figure S3. ^{1}H NMR spectrum of 1,2-bis(2-bromoethylthio)-4-methylbenzene in $CDCl_3$.

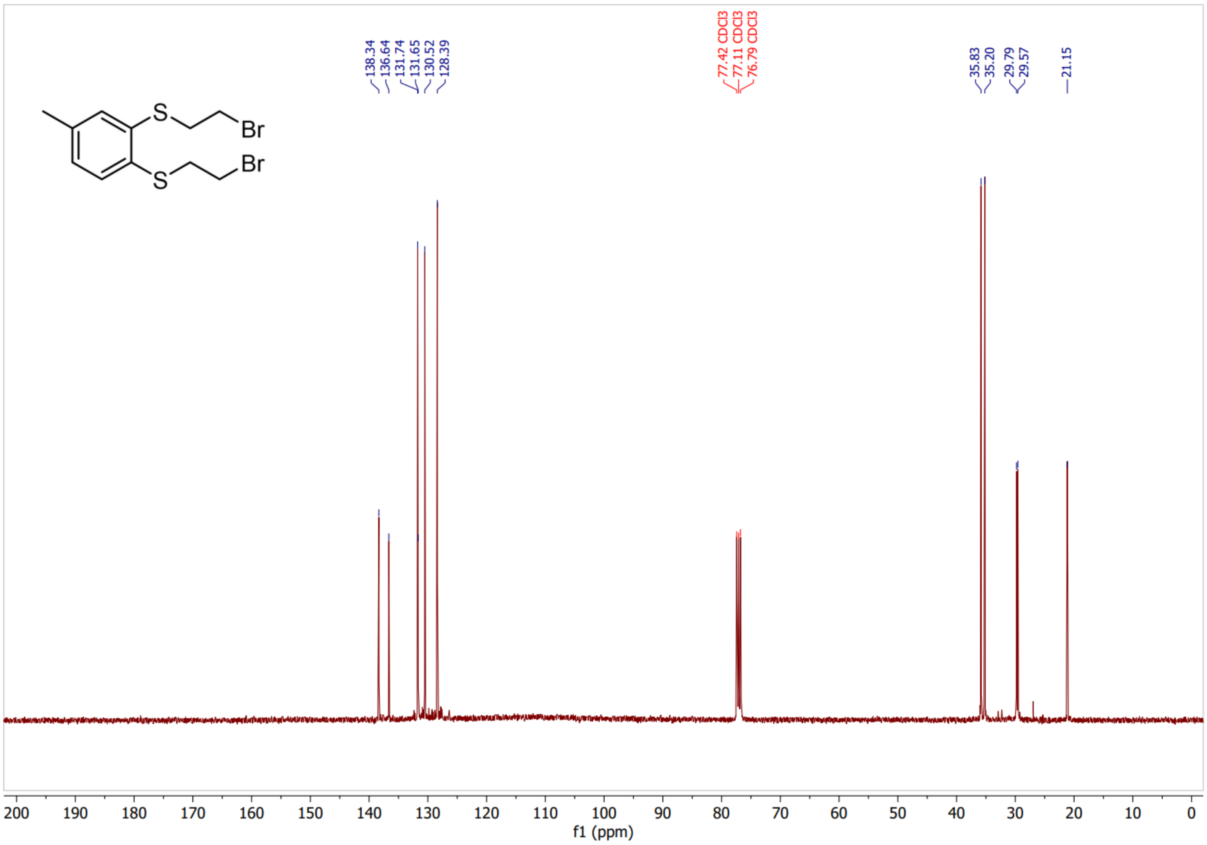

Figure S4. ^{13}C NMR spectrum of 1,2-bis(2-bromoethylthio)-4-methylbenzene in $CDCl_3$.

3. Thermogravimetry

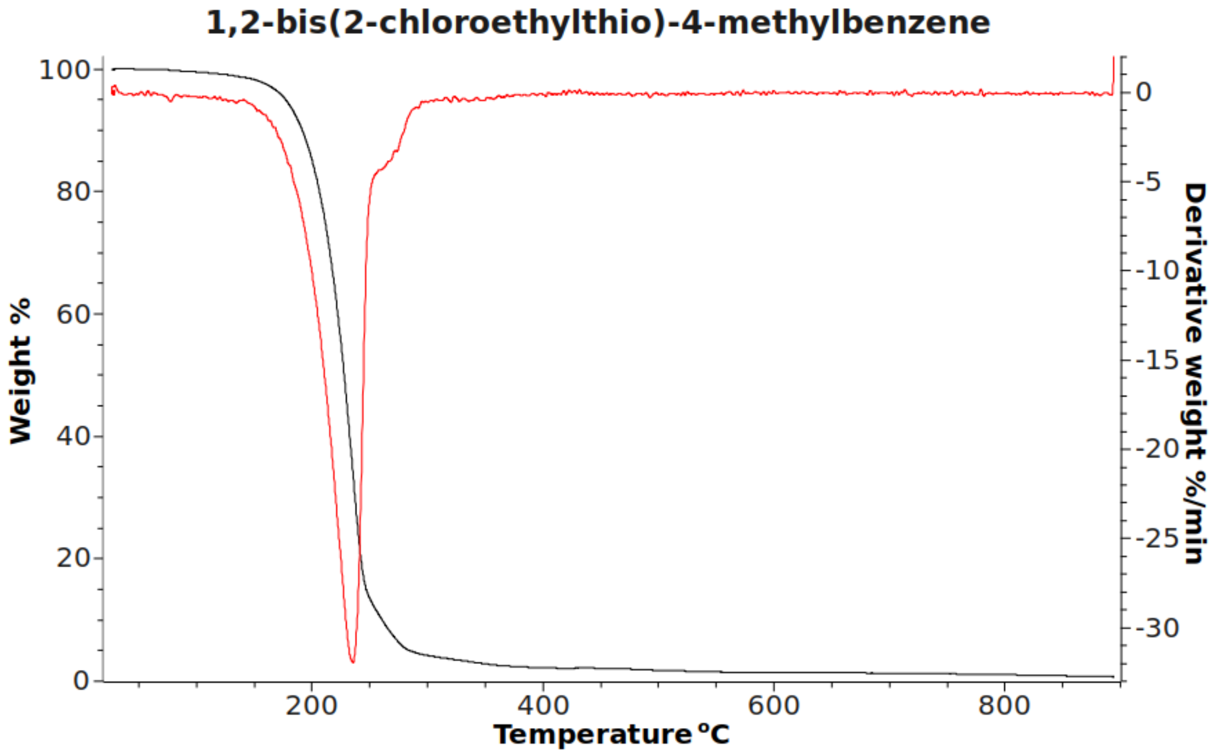

Figure S5. Weight losses and weight losses rate observed during the TGA measurements of compound **1**.

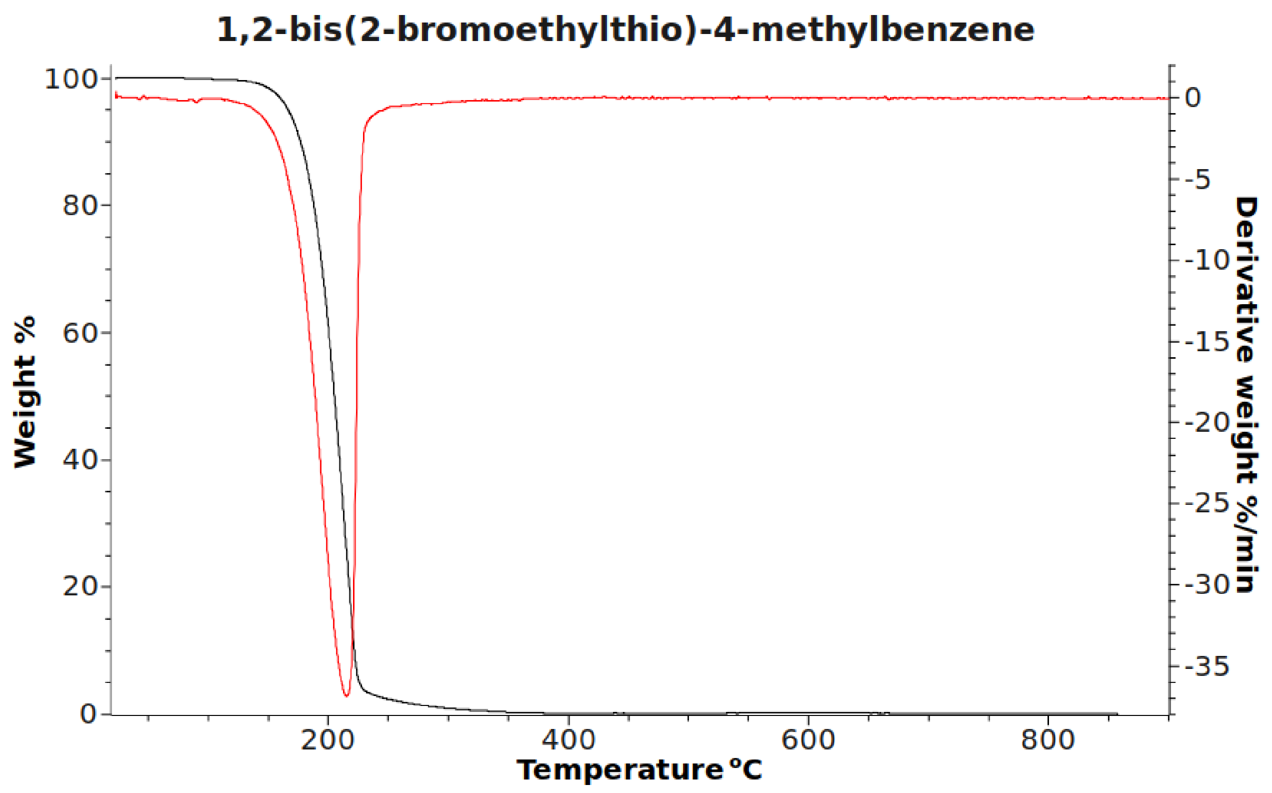

Figure S6. Weight losses and weight losses rate observed during the TGA measurements of compound **2**.

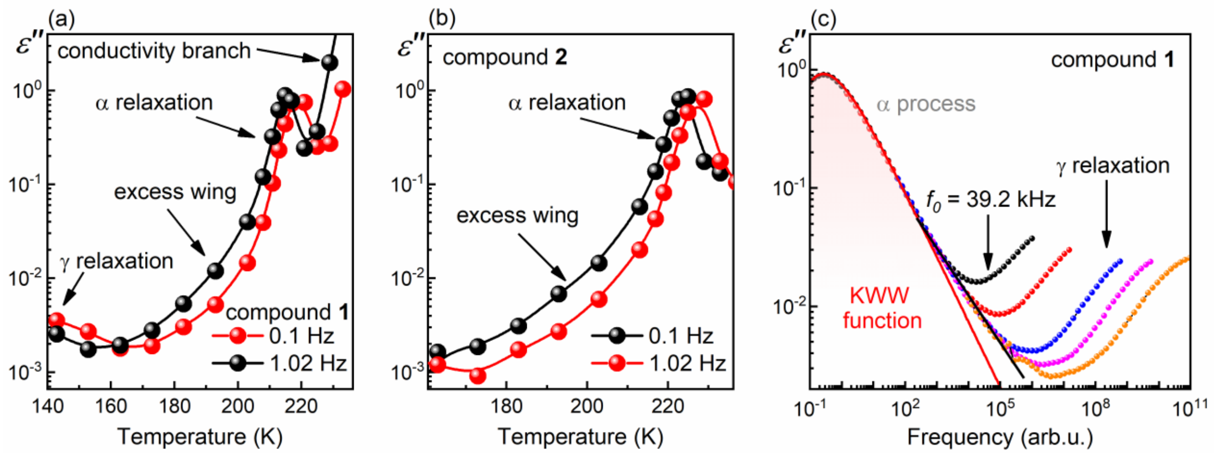

Figure S7. (a) Temperature dependence of ε'' at 0.1 Hz and 1.02 Hz for compound **1**. (b) $\varepsilon''(T)$ dependence at 0.1 Hz and 1.02 Hz for compound 2. (c) Master plot constructed for compound **2** by aligning the α-loss peak captured at 217 K with loss spectra recorded at lower temperatures. The red line represents the KWW fit function with $\beta_{KWW} = 0.55$, while the black line highlights the excess wing.

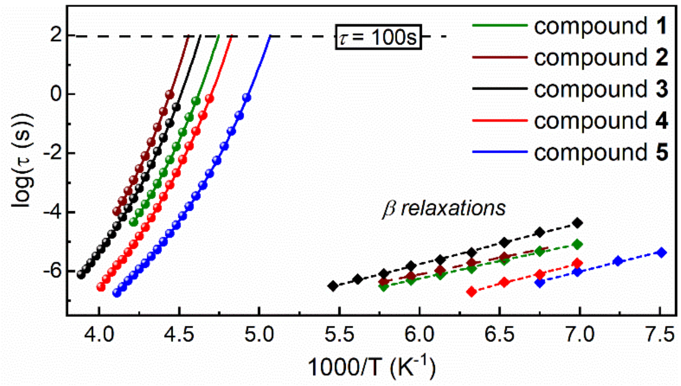

Figure S8. Temperature dependences of the main and secondary relaxation times for compounds **1**-**5**. The data for compounds **3**-**5** are reprinted from Ref. [11], with permission from Elsevier.

The relaxation times τ_β follow the Arrhenius law with the activation energies E_a equal to 23±2 kJ/mol for compound **1**, 22±2 kJ/mol for compound **2**, 27±2 kJ/mol for compounds **3** and **4**, and 26±2 kJ/mol for compound **5**. The temperature dependence of the main (α) relaxation times is similar across all compounds, closely following the Vogel–Fulcher–Tammann (VFT) law near the glass transition temperature, T_g. Notably, independently of the temperature conditions, the main relaxation of the acetyl ester (compound **3**) is faster compared to the bromine derivative **2**, yet slower compared to the chlorine derivative **1**. These differences in relaxation dynamics can be attributed to variations in molar mass: compound **3** has a lower molar mass (~328 g/mol) than compound **2** (~370 g/mol), but than compound **1** (~281 g/mol).

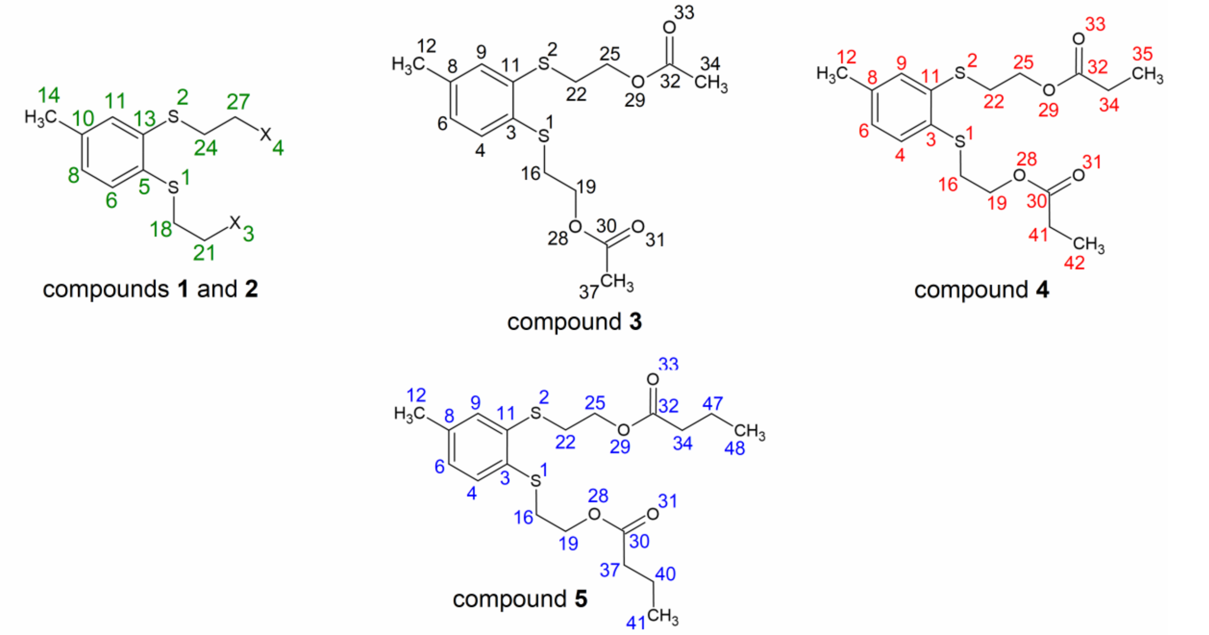

Figure S9. Chemical structure of compounds **1**-**5** with the adopted numbering scheme of non-hydrogen atoms.

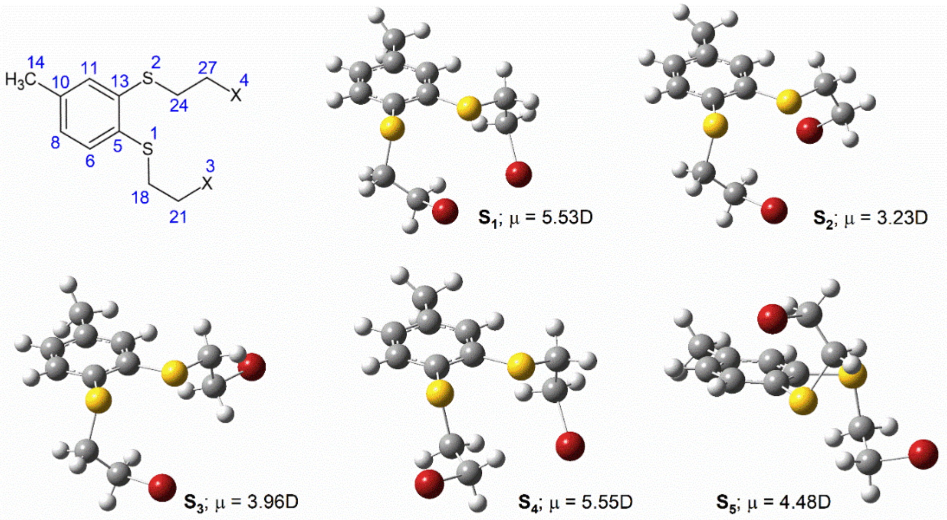

Figure S10. Chemical structure of compound **2** (X = Br) with the adopted numbering scheme of non-hydrogen atoms. The DFT-optimized representative conformers S_1–S_5 of compound **2** presented with the corresponding dipole moment values.

Table S1. The geometry of conformers S_1-S_5 in compounds **1** and **2**.

Dihedral angle	Conformer S_1		Conformer S_2		Conformer S_3	
	Compound 1	Compound **2**	Compound **1**	Compound **2**	Compound **1**	Compound **2**
13-5-1-18	-100.6°	-100.9°	-102.0°	-102.5°	-106.6°	-107.6°
5-1-18-21	88.4°	89.9°	76.8°	79.0°	76.4°	77.2°
1-18-21-3	69.0°	69.6°	70.8°	71.3°	70.4°	71.4°
5-13-2-24	167.6°	169.3°	174.5°	178.9°	174.4°	174.8°
13-2-24-27	-69.8°	-70.4°	-97.0°	-102.1°	-77.4°	-77.5°
2-24-27-4	-64.9°	-65.6°	70.8°	72.1°	179.3°	179.5°

Dihedral angle	Conformer S_4		Conformer S_5	
	Compound **1**	Compound **2**	Compound **1**	Compound **2**
13-5-1-18	-79.2°	-79.7°	68.6°	68.8°
5-1-18-21	161.7°	162.2°	59.2°	58.8°
1-18-21-3	66.1°	66.0°	65.9°	66.3°
5-13-2-24	162.9°	164.3°	94.5°	94.1°
13-2-24-27	-68.4°	-68.6°	-87.7°	-89.0°
2-24-27-4	-65.4°	-66.1°	-68.1°	-68.6°

Table S2. The geometry of conformers A_1, P_1, and B_1 of compounds **3**, **4**, and **5**.

Compound **3**- conformer A_1		Compound **4**- conformer P_1		Compound **5**- conformer B_1	
Dihedral angle	Value (°)	Dihedral angle	Value (°)	Dihedral angle	Value (°)
3-11-2-22	176.3	3-11-2-22	176.1	3-11-2-22	176.1
11-2-22-25	-77.3	11-2-22-25	-77.2	11-2-22-25	-77.2
2-22-25-29	-67.8	2-22-25-29	-67.6	2-22-25-29	-67.5
22-25-29-32	167.8	22-25-29-32	167.7	22-25-29-32	168.0
25-29-32-34	-178.7	25-29-32-34	-178.5	25-29-32-34	-178.4
11-3-1-16	-109.6	29-32-34-35	-178.8	29-32-34-47	-178.1
3-1-16-19	77.1	11-3-1-16	-109.5	32-34-47-48	-179.6
1-16-19-28	65.3	3-1-16-19	76.6	11-3-1-16	-109.6
16-19-28-30	80.4	1-16-19-28	65.2	3-1-16-19	76.5
19-28-30-37	-179.2	16-19-28-30	80.5	1-16-19-28	65.1
		19-28-30-41	-179.2	16-19-28-30	80.5
		28-30-41-42	-179.0	19-28-30-37	-179.2
				28-30-37-40	-179.1
				30-37-40-41	-179.8
